# Testosterone Deficiency Increases Hospital Readmission and Mortality Rates in Male Patients with Heart Failure

**DOI:** 10.5935/abc.20150078

**Published:** 2015-09

**Authors:** Marcelo Rodrigues dos Santos, Ana Luiza Carrari Sayegh, Raphaela Vilar Ramalho Groehs, Guilherme Fonseca, Ivani Credidio Trombetta, Antônio Carlos Pereira Barretto, Marco Antônio Arap, Carlos Eduardo Negrão, Holly R. Middlekauff, Maria-Janieire de Nazaré Nunes Alves

**Affiliations:** 1 Instituto do Coração (InCor) – Faculdade de Medicina da Universidade de São Paulo; 2 Universidade Nove de Julho (UNINOVE); 3 Faculdade de medicina da Universidade de São Paulo – Urologia; 4 Escola de Educação Física e Esporte da Universidade de São Paulo, São Paulo, SP – Brazil; 5 Division of Cardiology - David Geffen School of Medicine - University of California – USA

**Keywords:** Heart Failure / mortality, Testosterone / deficiency, Patient Readmission, Men

## Abstract

**Background:**

Testosterone deficiency in patients with heart failure (HF) is associated with decreased exercise capacity and mortality; however, its impact on hospital readmission rate is uncertain. Furthermore, the relationship between testosterone deficiency and sympathetic activation is unknown.

**Objective:**

We investigated the role of testosterone level on hospital readmission and mortality rates as well as sympathetic nerve activity in patients with HF.

**Methods:**

Total testosterone (TT) and free testosterone (FT) were measured in 110 hospitalized male patients with a left ventricular ejection fraction < 45% and New York Heart Association classification IV. The patients were placed into low testosterone (LT; n = 66) and normal testosterone (NT; n = 44) groups. Hypogonadism was defined as TT < 300 ng/dL and FT < 131 pmol/L. Muscle sympathetic nerve activity (MSNA) was recorded by microneurography in a subpopulation of 27 patients.

**Results:**

Length of hospital stay was longer in the LT group compared to in the NT group (37 ± 4 vs. 25 ± 4 days; p = 0.008). Similarly, the cumulative hazard of readmission within 1 year was greater in the LT group compared to in the NT group (44% vs. 22%, p = 0.001). In the single-predictor analysis, TT (hazard ratio [HR], 2.77; 95% confidence interval [CI], 1.58–4.85; p = 0.02) predicted hospital readmission within 90 days. In addition, TT (HR, 4.65; 95% CI, 2.67–8.10; p = 0.009) and readmission within 90 days (HR, 3.27; 95% CI, 1.23–8.69; p = 0.02) predicted increased mortality. Neurohumoral activation, as estimated by MSNA, was significantly higher in the LT group compared to in the NT group (65 ± 3 vs. 51 ± 4 bursts/100 heart beats; p < 0.001).

**Conclusion:**

These results support the concept that LT is an independent risk factor for hospital readmission within 90 days and increased mortality in patients with HF. Furthermore, increased MSNA was observed in patients with LT.

## Introduction

Symptoms attributable to heart failure (HF), including dyspnea, fatigue, and muscle weakness, lead to > 1 million hospitalizations per year in the United States and Brazil^1^, with readmission rates within 90 days approaching 50%^2,3^. Although the importance of HF-related costs as a major contributor to the healthcare spending crisis has been recognized, more than 20 pharmacological trials worldwide focusing on mortality as an endpoint have been negative^2^. As a large proportion of patients with HF would trade increased length of life for increased quality of life^4^, which is directly linked to exercise capacity^5^, it may be reasonable to refocus therapeutic targets in patients with HF to improve exercise and functional capacity.

Testosterone deficiency is recognized in a large number of male patients with advanced HF and is correlated with decreased functional class, exercise capacity, and muscle strength^6-8^, and in some studies^9^, but not all^10^, testosterone deficiency is associated with increased mortality. Testosterone therapy is recommended for men with a testosterone deficiency and symptoms of hypogonadism to increase exercise capacity. Testosterone therapy is associated with increased exercise tolerance in patients with HF compared with placebo, which is not explained by impaired cardiac function^8,11-14^.

Considering that cardiac function does not improve following testosterone therapy, and the growing acceptance of the "muscle hypothesis," which argues that exercise limitations in patients with chronic HF are focused on the periphery, including abnormalities in the reflexes activated during exercise^15,16^, testosterone therapy may have beneficial effects on the neurohumoral state in patients with HF. Indeed, decreased baroreceptor sensitivity and heart rate variability (HRV) have been reported in patients with a testosterone deficiency^17^.

The purpose of this study was to test the hypothesis that testosterone deficiency is associated with an increased risk for subsequent re-hospitalization within 30, 60, and 90 days. Additionally, we evaluated mortality rate in hospitalized male patients with HF due to decompensated HF. Moreover, we tested the hypothesis that patients with HF and testosterone deficiency have greater neurohumoral activation; i.e., increased levels of muscle sympathetic nerve activity (MSNA) compared to in patients with HF and normal testosterone (NT) levels to begin to understand the mechanisms underlying this potential inverse relationship.

## Methods

### Study Population

We prospectively evaluated 110 consecutive hospitalized male patients with HF who agreed to participate. Those who met the study inclusion criteria had acute decompensated HF and were functional class IV. Other study criteria were as follows: 1) age between 18 and 65 years old, 2) HF diagnosis > 6 months, and 3) left ventricular ejection fraction (LVEF) < 45%. Exclusion criteria were as follows: 1) history of coronary revascularization or myocardial infarction < 6 months before the study, 2) any hormonal treatment, including exogenous testosterone therapy, before or during the protocol, 3) advanced kidney disease, liver disease, or diabetes, 4) obesity (body mass index > 30 kg/m^2^), and 5) prostatic cancer or benign prostatic hyperplasia with or without anti-androgen therapy (finasteride, doxazosin, or tamsulosin). Written informed consent was obtained from all participants before the study; this study was approved by the local ethics committee and is registered at Clinical Trials (NCT01852994).

### Laboratory Measurements

For all patients, blood samples for androgen testing were collected on the morning of hospital admission. If the patient was admitted during the afternoon or at night, androgen testing was done the next morning. Serum levels of total testosterone (normal range, 300-965 ng/dL; intra-assay coefficient of variation [CV] ≤ 7.5% and inter-assay CV ≤ 5.4%), sex hormone-binding globulin (SHBG; normal range, 12-75 nmol/L; intra-assay CV ≤ 2.3% and inter-assay CV ≤ 6%) were determined according to standard laboratory techniques in the clinical laboratory at the Hospital da Clínicas, University of Sao Paulo Medical School. Free testosterone (normal range, 131-640 pmol/L) was calculated using SHBG and total testosterone in the formula proposed by Vermeulen et al^18^. Hypogonadism was defined in the laboratory as total testosterone < 300 ng/dL and free testosterone < 131 pmol/L^19,20^. B-type natriuretic peptide (BNP), C-reactive protein (CRP), hemoglobin, blood urea nitrogen (BUN), creatinine, sodium, potassium, and fasting blood glucose levels were also measured on the morning of hospital admission. Estimated glomerular filtration rate (eGFR) was calculated using the Modification of Diet in Renal Disease equation recommended by the National Kidney Disease Education Program.

### Muscle sympathetic nerve activity

Patients were stabilized on oral medication therapy and underwent microneurographic recording of MSNA. Microneurography of the peroneal nerve is a safe, precise, direct technique to record SNA directed to muscle^21^. MSNA was recorded in a subpopulation of 27 patients during hospitalization. In brief, a tungsten microelectrode was placed on the peroneal nerve, and a sympathetic neurogram was recorded. Nerve signals were amplified by 50,000-100,000 and band-pass filtered (700-2,000 Hz). Nerve activity for recording and analysis was rectified and integrated (time constant, 0.1 s) to obtain a mean voltage display. Muscle sympathetic bursts were identified by visual inspection (M.R.S.), who was blinded to the study protocol. MSNA was expressed as burst frequency (bursts/min) and burst incidence (bursts/100 heart beats [HB]).

### Echocardiography

LVEF was evaluated (Teicholz) using two-dimensional imaging according to standard methods^22^.

### Follow-up

All patients were followed up after discharge by a dedicated research nurse through electronic health records and by periodic phone calls.

### Statistical Analysis

The Kolmogorov-Smirnov test was used to verify that all data were normally distributed. Student's *t*-test and the Mann-Whitney *U*-test were used to compare parametric and non-parametric data, respectively. The chi-square (*X*^2^) test was used to analyze medications and the HF etiology data. The Cox proportional hazards model was used to test the associations between the analyzed variables and endpoints (readmissions within 30, 60, and 90 days and mortality). We included length of stay after the first hospital admission, age, LVEF, coronary artery disease (CAD) or no CAD, BNP, CRP, hemoglobin, urea, creatinine, eGFR, sodium, potassium, fasting blood glucose, total testosterone, free testosterone, and SHBG in the single-predictor analysis for readmission. We included the same variables as above in the single-predictor analysis for mortality, as well as readmission within 30, 60, and 90 days of discharge. Forward and backward stepwise multivariate model analyses (p = 0.10) were conducted to assess which factors independently predicted readmission and mortality in the single-predictor analysis. Survival was evaluated using a Kaplan-Meier analysis with 95% confidence intervals (CIs) between total testosterone and mortality within 1 year. Furthermore, a Kaplan-Meier analysis was used to evaluate the relationship between total testosterone level and readmission within 1 year. The Cox-Mantel log-rank test was used to evaluate differences in survival and readmission rates. A p value < 0.05 was considered statistically significant.

## Results

Sixty-six patients were classified with low testosterone (LT), and 44 had NT during hospitalization. The characteristics of the LT and NT groups are compared in Table 1. The LT and NT groups were not different in age, etiology of HF, LVEF, or medications used, but BNP, CRP, BUN, creatinine, and fasting blood glucose levels were significantly higher in the LT group than those in the NT group. In contrast, hemoglobin, eGFR, sodium, and total and free testosterone levels were significantly lower in the LT group than those in the NT group.

**Table 1:** Physical, clinical, and hormonal characteristics of men with heart failure at the first admission

**Variables**	**Low Testosterone (n = 66)**	**Normal Testosterone (n = 44)**	**p value**
Age, y	52 ± 1	51 ± 2	0.70
Weight, kg	72 ± 2	71 ± 2	0.52
Height, m	1.70 ± 0.01	1.69 ± 0.01	0.78
BMI, kg/m^2^	25 ± 1	24 ± 1	0.54
LVEF, %	25 ± 1	27 ± 1	0.24
TT, ng/dL	237 ± 11	652 ± 41	< 0.001
FT, pmol/L	124 ± 9	323 ± 21	< 0.001
SHBG, nmol/L	60 ± 3	73 ± 5	0.037
**Etiology of HF**			**0.81**
CAD	17 (26%)	9 (20%)	
Non CAD	49 (74%)	35 (80%)	
Chagasic	17	14	
Hypertensive	3	3	
Idiopathic	29	18	
**Treatment, N (%)**			
ACE-I/ARB	29 (44%)	24 (55%)	0.28
β-blockers	51 (77%)	26 (59%)	0.04
Diuretics	57 (86%)	33 (75%)	0.13
Digoxin	13 (20%)	10 (23%)	0.70
Statin	18 (27%)	8 (18%)	0.27
Aspirin	26 (39%)	11 (25%)	0.12
**Biomarkers**			
BNP, pg/mL	1725±153	804 ± 112	0.006
CRP, mg/L	35 ± 6	15 ± 3	0.007
BUN, mg/L	70 ± 4	56 ± 4	0.04
Creatinine, mg/L	1.66 ± 0.10	1.32 ± 0.05	0.02
Fasting glucose, mg/L	114 ± 3	94 ± 2	0.01
Hemoglobin, g/dL	12.30 ± 0.26	13.57 ± 0.30	0.007
GFR, mL min^-1^ 1.73 m^-2^	49 ± 2	55 ± 1	0.03
Sodium, mEq/L	135 ± 1	137 ± 0.4	0.04
Potassium, mEq/L	4.38 ± 0.07	4.51 ± 0.08	0.29

Six patients in the low testosterone group and two patients in the normal testosterone group died during the first admission and were excluded from subsequent analyses. BMI: body mass index; LVEF: left ventricular ejection fraction; TT: total testosterone; FT: free testosterone; SHBG:sex hormone-binding globulin; CAD: coronary artery disease; ACE-I/ARB: angiotensin-converting enzyme inhibitor/angiotensin receptor blocker; BNP: B-type natriuretic peptide; CRP: C-reactive protein; BUN: blood urea nitrogen; GFR: glomerular filtration rate.

### Length of hospital stay during the first admission

**Figure 1:**
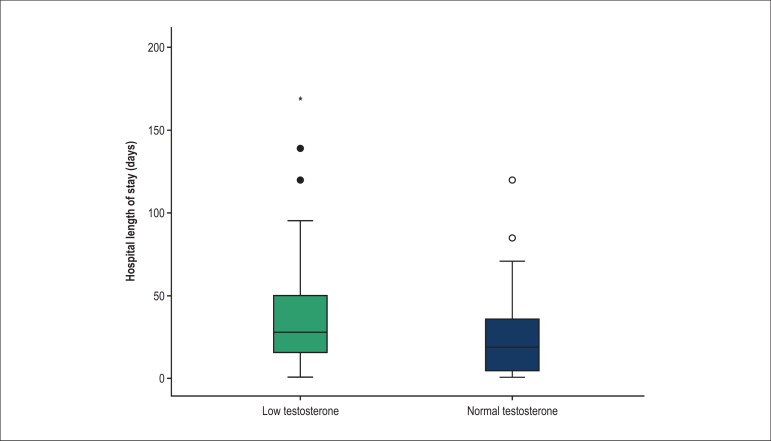
Length of hospital stay in patients with heart failure and low testosterone (LT; n = 66) and normal testosterone (NT; n = 44). The LT group had a longer hospitalization stay than the NT group at the first admission. ^*^Between group difference, p = 0.008.

The LT group had a significantly longer hospital stay than the NT group (37 ± 4 vs. 25 ± 4 days, respectively) (Figure 1; p = 0.008). This relationship persisted even when outliers (values >2 standard deviations) were excluded. Six patients died in the LT group and two died in the NT group during this first admission.

### Readmissions after the first admission

**Figure 2:**
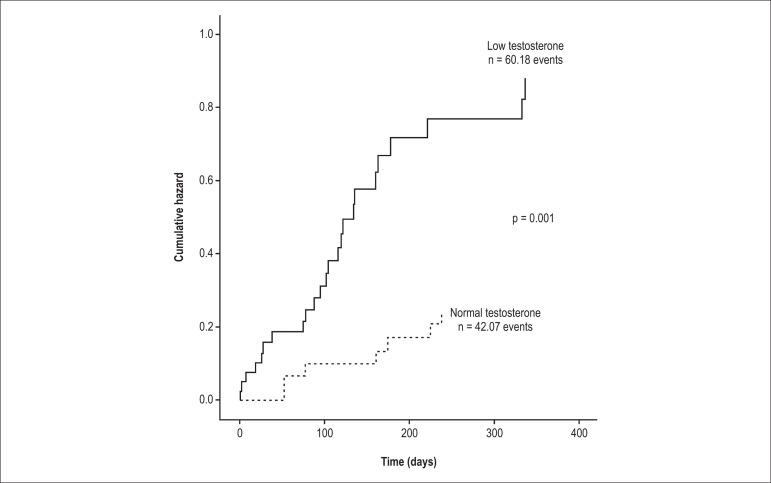
Kaplan–Meier readmission curves within the 1-year follow-up in patients with heart failure. The low testosterone group showed more cumulative readmissions than the normal testosterone group within the year (p = 0.001). The 1-year follow-up started at hospital discharge (time zero).

The medications at discharge were not different between the LT and NT groups (Table 2). The median times for the first readmission were 94 days (LT group) and 137 days (NT group). Readmission rate was greater in the LT compared to in the NT group when measured at 30, 60, and 90 days. However, readmission rates within 30 and 60 days were not related to any variable tested in the Cox regression. Total testosterone was the only variable related to this poor outcome in the single-predictor analysis for readmission within 90 days (hazard ratio [HR], 2.77; 95% CI, 1.58-4.85, p = 0.02; Table 3). All variables related to readmission within 90 days in the single-predictor analysis were included in the multivariate Cox proportional hazard model analyses. The final model after forward and backward stepwise analyses consisted of only total testosterone (forward: HR, 2.49; 95% CI, 1.33-4.65; p = 0.04 and backward: HR, 2.48; 95% CI, 1.33-4.65; p = 0.004) for readmission within 90 days. In addition, the cumulative hazard for readmission was higher in the LT group compared to in the NT group (Figure 2; n = 102; p = 0.001) at the 1-year follow-up.

**Table 2:** Medications and dally dosages at discharge (first admission) In men with heart failure

**Variables**	**Low Testosterone (n = 60)**	**Normal Testosterone (n = 42)**	**p value**
**Treatment, N (%)**			
β-blocker	52 (87%)	39 (93%)	NS
ACE-I/ARB	41 (68%)	36 (86%)	NS
Spironolactone	25 (42%)	19 (45%)	NS
Diuretics	43 (72%)	34 (81%)	NS
Digoxin	10 (17%)	15 (36%)	NS
Hydralazine	36 (60%)	20 (48%)	NS
Statin	22 (37%)	8 (19%)	NS
Aspirin	19 (32%)	10 (24%)	NS

Six patients in the low testosterone group and two patients in the normal testosterone group died during the first admission. ACE-I/ARB: angiotensin-converting enzyme inhibitor/angiotensin receptor blocker; NS: not significant.

**Table 3:** Single-predictor models of the Cox proportional hazard analysis for readmission within 90 days and mortality. Low testosterone group (n = 60) and normal testosterone group (n = 42)

**Readmission within 90 days**	**Hazard ratio**	**95% CI**	**p value**
Total testosterone, ng/dL	2.77	1.58-4.85	0.02
**Mortality**	**Hazard ratio**	**95% CI**	**p value**
Sodium, mEq/L	0.89	0.81-0.97	0.01
Total testosterone, ng/dL	4.65	2.67-8.10	0.009
Readmission - 90 days	3.27	1.23-8.69	0.02

Six patients in the low testosterone group and two patients in the normal testosterone group were excluded from the single-predictor models due to mortality during the first admission. CI: confidence interval.

### Mortality at the 1-year follow-up

**Figure 3:**
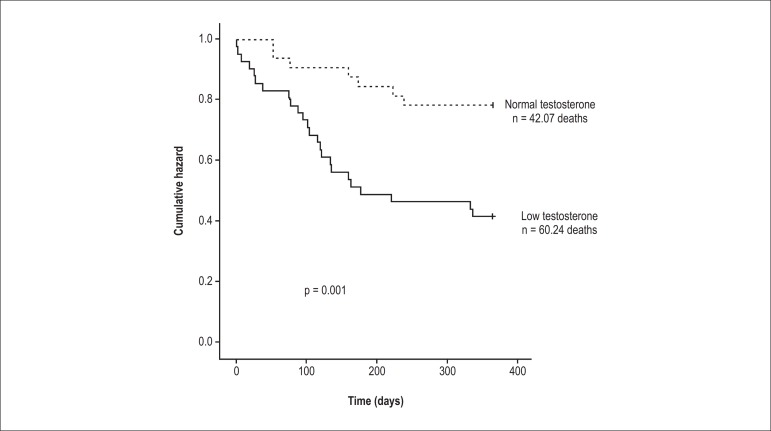
Kaplan–Meier survival curves within the 1-year follow-up in patients with heart failure. The low testosterone group had a higher mortality rate than the normal testosterone group within the year (p = 0.001). The 1-year follow-up started at hospital discharge (time zero).

Following first discharge, the LT group had significantly higher mortality compared than the NT group (Figure 3; n = 102; p = 0.001) at the 1-year follow-up: 24 patients in the LT group and seven in the NT group died within 1 year after hospital discharge. In the single-predictor analysis, total testosterone (HR, 4.65; 95% CI, 2.67-8.10; p = 0.009; Table 3) and readmission within 90 days (HR, 3.27; 95% CI, 1.23-8.69; p = 0.02; Table 3) were predictors of mortality. All variables that were related to mortality in the single-predictor analysis were included in the multivariate Cox proportional hazard model analyses. The final model after the forward stepwise analysis included total testosterone (forward: HR, 3.86; 95% CI, 2.06-7.21; p < 0.001) and readmission within 90 days (forward: HR, 3.08; 95% CI, 1.57-6.04; p = 0.001) for mortality. The backward stepwise analysis for mortality included total testosterone (HR, 3.86; 95% CI, 2.06-7.21; p < 0.0001), eGFR (HR, 0.97; 95% CI, 0.95-0.9; p = 0.034), readmission within 90 days (HR, 2.39; 95% CI, 1.05-5.45; p = 0.038), and readmission within 60 days (HR, 2.96; 95% CI, 1.01-8.67; p = 0.047).

### Muscle sympathetic nerve activity

MSNA was significantly higher in the LT group compared to in the NT group when calculated as bursts/100 HB, (65 ± 3 vs. 51 ± 4 bursts/100 HB, p < 0.001). MSNA tended to be higher in the LT group compared to in the NT group when calculated as bursts/min (47 ± 3 vs. 43 ± 4 bursts/min, p > 0.05), but was not statistically significant.

## Discussion

Although testosterone deficiency is associated with heart disease and elevated mortality^23^, testosterone level is not commonly used in clinical practice to measure the severity and prognosis in patients with HF. Our findings extend the observation that LT is associated with an increased cardiac risk by demonstrating that LT is associated with increased mortality in a cohort with HF. Furthermore, deficiency in total testosterone was an independent risk factor for increased morbidity as evidenced by the increase in hospital readmissions within 90 days.

The stepwise analysis for mortality was strongly related to readmission within 90 days (forward) and readmission within 60 days (backward), as well as total testosterone and eGFR. One review reported that later hospital readmission (> 30 days) is associated with renal insufficiency and elevated BNP level^2^. Interestingly, we did not find an association between earlier readmission (within 30 days) and a testosterone deficiency in our patients. This result is consistent with the notion that a 30-day readmission may be related to poor social support and low socioeconomic status rather than to an increase in clinical comorbidities^2^. In fact, we found that a testosterone deficiency was associated with greater activation of the sympathetic nervous system, and increased sympathetic nerve activity is a risk factor for increased mortality in patients with HF^24^. The increased sympathetic activation may provide a potential mechanism, which will be discussed below.

Some studies have reported an inconsistent association between testosterone and mortality in patients with HF. Jankowska et al^9^ found that low total testosterone is associated with increased mortality. In contrast, Guder et al^10^ reported that LT is not an independent risk factor for decreased survival in patients with HF. These disparate findings can be explained by differences in the HF populations; the population studied by Guder et al^10^ included a large proportion (~50%) of patients with HF and preserved LVEF, unlike those in the study by Jankowska et al^9^ In addition, they speculated that their findings differed from those of Jankowska et al. because they included additional prognostic variables, such as medication use, inflammatory markers, and others. Importantly, the additional variables included by Guder et al^10^ as well as several others were included in our study, and LT remained an independent predictor of increased mortality in patients with advanced HF and systolic dysfunction in our study.

We found that total testosterone, not free testosterone, was associated with poor outcomes. Total testosterone consists of free testosterone plus testosterone bound to SHBG and albumin; free testosterone is the active fraction. A meta-analysis reported that a decrease in total testosterone is associated with 35% and 25% increased risks of all-cause and cardiovascular disease mortality in men, respectively^25^. Another study on patients with HF showed that a deficiency in SHBG is associated with a higher risk of cardiac death^26^. In contrast, high levels of SHBG lead to a lower bioavailability of free testosterone and has been linked to increased mortality in men when augmented by SHBG^27^. Although we found a significant difference in SHBG levels between the LT and NT groups, the result remained within the normal range in our laboratory (20.6-76.7 nmol/L). SHBG levels can be altered by non-cardiac medications, diet, and specific illnesses but the relative importance of these factors in our patients is not known.

Although a deficiency in androgens has been previously associated with increased morbidity, particularly a decrease in exercise capacity, our study is the first to examine the association between LT and hospital readmissions, a critically important endpoint in this era of skyrocketing medical costs. The patients with HF and LT had similar LVEF, New York Heart Association functional class, HF etiology, and discharge medications, as those of patients with HF and NT. Interestingly, LT was accompanied by several markers of increased HF severity, including higher BNP and CRP levels, more severe renal dysfunction, and anemia, yet testosterone deficiency remained an independent predictor of readmission within 90 days of hospital discharge.

It is unknown if a testosterone deficiency is only a strong marker for poor prognosis in patients with HF, or whether LT contributes directly to increased morbidity and mortality. Testosterone deficiency is associated with many signs and symptoms, several of which are indistinguishable from normal aging, including declining libido, increased body fat, osteoporosis, mild anemia, depression, and fatigue^28^. The most easily recognizable manifestation of a testosterone deficiency is a decline in muscle strength and bulk, which is associated with a decline in exercise capacity^28^. Exogenous testosterone improves exercise capacity^29,30^. Baseline testosterone levels were directly related to exercise capacity in five small, controlled trials of testosterone treatment in patients with HF, including those with LT, NT, and one trial of women with HF. Furthermore, 3-month testosterone therapy compared with placebo is associated with increased exercise capacity in many studies^8,11-14^. As hospitalization for HF is often prompted by increased fatigue and shortness of breath and the mechanisms underlying these symptoms vary^31-33^ but includes progressive muscle weakness, it is tempting to speculate that testosterone deficiency may be an important contributor to the ongoing peripheral muscle decline, and thereby is a direct contributor to re-hospitalization risk. A controlled trial of testosterone therapy in patients with advanced HF would clarify this issue.

According to the muscle hypothesis, exercise limitations in patients with HF reside in the periphery, specifically in skeletal muscles^15,16,34,35^. Furthermore, skeletal muscle abnormalities in patients with HF may contribute to abnormal neurohumoral activation, including exaggerated increases in sympathetic nerve activity^35-38^. In a study of male rats with HF, castration was associated with changes in cardiac sympathetic nerve activity and increased plasma norepinephrine levels^39^. This sympatho-excitation was reversed by testosterone replacement therapy, as observed by decreased plasma norepinephrine, increased myocardial norepinephrine, the density of tyrosine hydroxylase (TH) protein-labeled nerve fibers, and upregulated expression of myocardial TH protein. In a recent study of patients with mild HF, Rydlewska et al^17^ reported that testosterone deficiency is directly related to lower baroreceptor sensitivity and HRV. Similarly, castration significantly attenuates baroreceptor control of reflex bradycardia versus no effect on reflex tachycardia in rats. Testosterone replacement increases baroreflex sensitivity and restores reflex bradycardic responses^40^. In our study, resting MSNA was higher in patients with LT; it is intriguing to speculate that testosterone therapy may have a modulating effect on sympathetic activation in patients with HF, which may be mediated by ameliorating abnormal reflexes originating in peripheral muscles and/or potentially through an effect on the baroreceptors themselves.

## Limitations

We recognize several limitations in our study. Androgen levels were only measured at one time point during the index admission for decompensated HF. It is not known whether androgen levels fluctuate in patients with HF based on their clinical status. All patients with HF were decompensated at the time testosterone levels were obtained; it is unknown whether testosterone provides similar important prognostic information in less ill patients with HF.

MSNA was recorded in 25% of the patients during hospitalization after they were stabilized because we could not conduct this procedure during acute decompensation. Our MSNA results are representative of only a small subgroup of patients; thus, the results need to be confirmed in a larger group.

We did not have exercise data on our patients, and peak VO_2_ is known to provide important prognostic information in patients with advanced HF. However, all patients were decompensated at the time of enrollment, and all were too ill to perform a CPX, which is a uniformly poor prognostic sign. Importantly, within this group of patients with HF who were too ill to provide exercise data, testosterone levels provided important prognostic information. The incidence of sleep apnea was not assessed in this group, which is another important risk factor that should be assessed in the future.

Finally, this study was conducted at a single institution (Heart Institute at the University of São Paulo, Brazil) and we only included males with HF. It is unknown whether testosterone therapy would be beneficial in females with HF, but it is an intriguing possibility.

## Conclusion

Our results support the notion that LT is an independent risk factor for hospital readmission within 90 days and increased mortality in patients with HF. Furthermore, we observed a possible modulating effect of LT on sympathetic activation in patients with HF.
